# Monomethylation of cytosolic 5′-nucleotidase 1A affects its recognition by inclusion body myositis autoantibodies

**DOI:** 10.1177/22143602261487219

**Published:** 2026-09-02

**Authors:** Fleur Brinkman, Marian Barandica Graciani, Amrah Weijn, Filine Swets, Seher Al Bana, Chien-Yun Lee, Ger J. M. Pruijn

**Affiliations:** 1 Department of Biomolecular Chemistry, Institute for Molecules and Materials, 226184 Radboud University, Nijmegen, The Netherlands; 2 School of Life Sciences, Technical University of Munich, Freising, Germany

**Keywords:** protein accumulation, inclusion body myositis, monomethylation, autoantibodies, cytosolic 5′-nucleotidase 1A

## Abstract

The progressive muscle disease inclusion body myositis (IBM) is characterized, amongst others, by inflammatory features and protein accumulation in skeletal muscle fibers. One of the proteins that accumulates in perinuclear and peripheral regions of muscle fibers is cytosolic 5′-nucleotidase 1A (cN1A), the target of anti-cN1A autoantibodies, which are found in many IBM patients. To shed more light on potential pathogenic aspects of IBM, we identified post-translational modifications of cN1A in human skeletal muscle. Immunoaffinity-purification followed by mass spectrometry resulted in the identification of three monomethylation sites, K178, R223, and K243. Single amino acid substitutions of each of these methylation sites did not detectably affect the accumulation of cN1A in perinuclear regions of cultured human cell lines. Two of these monomethylation sites, R223 and K243, are located within one of the previously identified major linear epitope regions of cN1A. Synthetic peptides, corresponding to aa 219 – aa 247, were used to investigate the effect of monomethylation on the antigenicity of this epitope by ELISA. The results showed that the simultaneous monomethylation of R223 and K243 may enhance its recognition by IgG autoantibodies. We conclude that cN1A contains at least three amino acids that can be monomethylated and that monomethylation may affect its autoantigenicity.

## Introduction

The progressive inflammatory myopathy inclusion body myositis (IBM) is most prevalent in men over 45 years old.^1^ IBM is characterized by degenerative features in patients’ muscle fibers, including mitochondrial degeneration, rimmed vacuoles, and the accumulation of proteins such as TDP-43, amyloid-beta, p62, and tau in specific areas of the muscle fibers.^2,3^ Also, inflammatory characteristics are commonly observed in patients’ muscles, including the infiltration of CD8-positive T-cells and the upregulation of MHC class I expression.^1^ In addition, serological changes have been observed in IBM, such as mild upregulation of creatine kinase levels^4,5^ and the presence of anti-cN1A autoantibodies.^5^ Anti-cN1A autoantibodies are present in 29%-80% of IBM patients, and, albeit at lower frequencies, also in the sera of patients with dermatomyositis, polymyositis, systemic lupus erythematosus, and Sjögren’s syndrome.^6–8^ IBM patients with anti-cN1A autoantibodies were reported to have more respiratory problems^9^ and a higher mortality rate.^10^ However, several other studies have not found correlations between the presence of anti-cN1A autoantibodies and IBM disease severity.^6,11–13^ cN1A (cytosolic 5′-nucleotidase 1A) catalyzes the dephosphorylation of AMP, which is important for purine nucleotide metabolism. cN1A is most highly expressed in muscle tissue.^14,15^ In muscle fibers of IBM patients, cN1A has been reported to accumulate in the perinuclear region,^6,16–18^ around rimmed vacuoles,^6,16,17^ near sarcoplasmic membranes, and in the sarcoplasm.^18^ Besides the accumulations in patient muscle fibers, ectopically expressed cN1A also appeared to accumulate in cultured cells, more specifically in filaments in the perinuclear region.^19^

The accumulations formed by cN1A in cultured cells are reminiscent of those formed by other enzymes involved in nucleotide metabolism, such as CTP synthase (CTPS) and inosine-5′-monophosphate dehydrogenase (IMPDH). Under certain circumstances, these proteins form so-called rods and rings structures (or cytoophidia). It has been found that posttranslational modifications (PTMs) can regulate the formation of CTPS filaments. CTPS forms tetramers, which stack to create bundles, a process controlled by methylation of an arginine residue.^20^ Moreover, ubiquitination has been reported to regulate the dynamic bundling of smaller CTPS bundles into larger ones.^21^

PTMs are also involved in disease-associated protein accumulation. In Alzheimer’s disease (AD), for example, neuronal filaments play an important pathological role,^22^ which involves phosphorylated, ubiquitinated, acetylated, and methylated tau. Different PTM patterns can be distinguished for patients with AD compared to healthy controls.^23^ The hyperphosphorylation^24^ and ubiquitination^25^ of tau have been shown to lead to misfolding and accumulation. Monomethylation of soluble tau may alter its subcellular location,^24^ and acetylated tau has pathogenic properties by compromising the cytoskeletal machinery.^26^ Thus, many PTMs on tau lead to misfolding, accumulation, and translocation, thereby contributing to the pathology of AD.

Protein accumulation has been shown to play an important role in various diseases. A better understanding of the formation of such accumulations, including the role of PTMs, provides more insight into the etiology and pathogenesis of these diseases. Here, we studied the modification state of cN1A in skeletal muscle to gain more insight into molecular features important for the formation of cN1A-containing accumulations, which ultimately may lead to a better understanding of this phenomenon in relation to IBM. Previously, tyrosine phosphorylation has been reported for cN1A in human ovarian cancer tissue^27^ and in a mammalian neuroblastoma cell line.^28^ Since cN1A is most highly expressed in the muscle, and particularly muscle tissue is affected in IBM, we isolated cN1A from human skeletal muscle to investigate its modification status. In addition, the effects of several PTMs identified on cN1A accumulation and its recognition by autoantibodies were analyzed as well.

## Materials and methods

### Preparation of muscle lysate

Preparation of muscle lysate and immunoaffinity purification were performed as described previously, with small adjustments.^7^ Healthy human hamstring muscle tissue obtained from reconstructive surgery (kind gift from Dr. Snoeck, Canisius-Wilhelmina Hospital, Nijmegen, the Netherlands) was frozen in liquid nitrogen and stored at -80°C. Pieces of muscle tissue were pulverized with a Microdismembrator (Braun Biotech International, Melsungen, Germany), resuspended in lysis buffer (50 mM Tris-HCl, pH 7.5, 150 mM sodium chloride, 1% Nonidet P-40 (NP-40), 0.5% sodium deoxycholate (DOC), 0.1% sodium dodecyl sulfate (SDS), 1x Complete protease inhibitor (Roche), 0.5 mM phenylmethylsulfonyl fluoride (PMSF), and kept on ice for at least 10 minutes. The lysates were centrifuged for 10 minutes at 14,000 x g, at 4°C. The protein concentration in the supernatant was determined by a bicinchoninic acid (BCA, Thermo Scientific) assay. The supernatant was aliquoted, flash-frozen in liquid nitrogen, and kept at -80 °C until further use.

### Immunoaffinity purification

Three sera were used for the isolation of cN1A from muscle lysate: two from IBM patients known to have anti-cN1A autoantibodies and one from a healthy person negative for anti-cN1A autoantibodies. Magnetic protein A agarose beads (50% slurry; Pierce), 200 μL, were washed twice with PBS and incubated under agitation with 200 μL serum for 1 hour at room temperature (RT). The beads were washed twice with PBS. Antibodies bound to the beads were covalently coupled to protein A on the beads by incubation with 20 mM dimethyl pimelimidate dihydrochloride (DMP) in 0.2 M sodium borate buffer, pH 9.0, under agitation for 30 min at room temperature. Subsequently, the beads were washed three times with freshly prepared 0.2 M ethanolamine, pH 8.0, twice with PBS, and twice with lysis buffer. Muscle lysate (35 mg protein) was pre-incubated with 200 μL of magnetic protein A agarose beads (50% slurry; Pierce) for 1 hour at RT to remove endogenous IgG from the muscle lysate. The IgG-depleted muscle lysate was incubated overnight with the serum antibody-coupled protein A agarose beads at 4 °C on an end-over-end rotator. Subsequently, the beads were washed five times with lysis buffer at 4 °C. The bound proteins were eluted in SDS sample buffer (2% SDS, 5% β-mercaptoethanol, 10% glycerol, 0.01% bromophenol blue, 125 mM Tris-HCl, pH 6.8) and heated at 95 °C for 10 minutes. A Western blot was performed to confirm the results.

### Sample preparation for mass spectrometry

Eluted proteins were loaded on a 12% SDS-PAGE gel. The gel was stained with colloidal Coomassie staining overnight under agitation at RT. The gel was destained with demineralized water. A piece of gel containing polypeptides was excised from the gel at around 44 kDa. The gel pieces were cut into smaller pieces. All washing and incubation steps were done for 5 min at RT unless otherwise specified. Subsequently, the pieces of gel were washed three times with 50 mM ammonium bicarbonate, 50% acetonitrile, and 100% acetonitrile and incubated with 10 mM DTT for 20 min at 56 °C. After washing with 100% acetonitrile, the gel pieces were incubated with 50 mM 2-chloroacetamide in 50 mM ammonium bicarbonate for 20 min in the dark. The gel was washed with 100% acetonitrile and twice with 50 mM ammonium bicarbonate, followed by 100% acetonitrile. Trypsin (Promega) was added at a concentration of 12.5 ng/μL in 50 mM ammonium bicarbonate until the gel pieces remained covered with liquid. Digestion was done overnight at 37 °C. After brief centrifugation to collect all condensed buffer, 2% trifluoroacetic acid was added, followed by shaking for 20 min. The supernatant was transferred to a clean tube. Formic acid in acetonitrile (0.1%) was added to the gel pieces and after incubation for 20 min under agitation, the supernatant was added to the previously collected supernatant. The samples were dried completely using a SpeedVac.

### LC-MS/MS analysis

Twenty-five percent of the gel-extracted peptides were reconstituted in 0.1% formic acid (FA) for analysis. The samples were processed using an UltiMate 3000 HPLC system interfaced with an Orbitrap Fusion Lumos mass spectrometer (Thermo Fisher Scientific). During sample loading, a drawing speed of 0.5 µL/s was maintained. Peptides were initially captured on a self-packed trap column (ReproSil-pur C18-AQ, 5 µm, 20 mm × 75 µm; Dr. Maisch) using solvent A (0.1% FA in H_2_O) at a 5 µL/min flow rate. Subsequent separation was achieved on a 50 cm analytical column (ReproSil Gold C18-AQ, 3 µm, 75 µm ID, self-packed; Dr. Maisch) at 300 nL/min. The elution utilized a 50-minute linear gradient increasing from 4% to 32% solvent B (0.1% FA and 5% DMSO in acetonitrile). Mass spectrometry was conducted in data-dependent acquisition (DDA) mode with a 2-second cycle time. Full MS1 scans were recorded in the Orbitrap (range 360–1,300 m/z) at 60,000 resolution, utilizing a 100% AGC target and a maximum injection time (maxIT) of 118 ms. For MS2 analysis, precursor ions with charges between 2+ and 6+ were prioritized, with dynamic exclusion enabled for 30 seconds. Higher-energy collisional dissociation (HCD) was employed for fragmentation at a 30% normalized collision energy (NCE). MS2 spectra were captured at 15,000 resolution with a 1.3 m/z isolation window, 150% AGC target, and 22 ms maxIT.

### Database search

The resulting raw data were processed through FragPipe v23 ^26^ (MSFragger v4.3 and IonQuant v1.11.11). Searches were performed against the Swiss-Prot human reference database (downloaded April 19, 2022; 20,435 sequences). Proteolytic digestion was defined by strict trypsin specificity, permitting a maximum of three missed cleavages. A 1% False Discovery Rate (FDR) filter was applied at both the PSM and protein levels via PeptideProphet. For open database searching, standard default parameters were applied.^29^ Closed searches incorporated variable modifications for lysine and arginine monomethylation (+14.0156 Da), with a limit of two modifications per peptide sequence. The identification of monomethylated cN1A peptides was manually validated by MS2 spectral inspection using the Interactive Peptide Spectral Annotator.^30^ The occurrences of the monomethylated peptides were calculated by dividing the intensity of the monomethylated peptide by the intensity of the monomethylated peptide plus the intensity of the non-methylated peptide.

### Cloning

The human cN1A cDNA was inserted into the pEqualizer-L plasmid (Addgene plasmid # 169732) with the EGFP sequence fused to the 5′-end. This plasmid was used to create mutant cN1A expression plasmids with single amino acid substitutions at modification sites. The lysines at positions 178 and 243 were changed to arginine, and the arginine at position 223 was mutated to lysine by a PCR-based approach. Primers were (mutations in italic): K178R (5′-GAA*CGA*GTG​CGA​GAA​GCC​ATT​GAT​GAG​GGG-3′ and 5′-TCG​CAC*TCG*TTC​CGC​ATC​GGC​TGA​CAA-3′), R223K (5′-TCG​GAG*AAG*ATC​GTC​AAG​GCC​CAC​GGG​CTG-3′ and 5′-GAC​GAT*CTT*CTC​CGA​CTC​GTC​CGA​GAA​GAG-3′), and K243R (5′-AAC*CGG*CCT​CTG​GCT​CAG​GGC​CCC​TTA-3′ and 5′-CAG​AGG*CCG*GTT​CTC​GTG​GGC​CTT​CTC​ATG-3′).

### Tissue culture

HEp-2 cells were passaged twice a week to keep the confluency below 80%. Cells were maintained at 37 °C in a humidified incubator with 5% CO_2_ in Dulbecco’s modified Eagle’s medium (DMEM), high glucose, GlutaMAX™-I, and pyruvate (Gibco™, Thermo Scientific), supplemented with 10% (v/v) fetal bovine serum (Sigma-Aldrich) and penicillin-streptomycin (Gibco).

### Transfection

Cells were seeded the day before transfection on coverslips coated with poly-L-lysine. For the transfection, 2 µL of Lipofectamine 2000 was used for 800 ng of DNA. Medium was replaced 4-6 hours after transfection.

### Immunofluorescence

Cells were fixed with 4% paraformaldehyde 48h after transection. Before and after fixation, cells were washed three times with PBS. Fixed cells were kept in PBS at 4 °C in the dark until staining. Cells were permeabilized with 0.1% Triton X-100 in PBS for 20 minutes. After washing three times with PBS, DNA was stained with DAPI (1 µg/mL) for 10 min. Cells were washed twice with PBS before mounting the coverslips on microscopy slides with fluorescence Mounting Medium (Dako). Cells were imaged using a fluorescence microscope (EVOS M5000, Thermo Scientific), using 40x and/or 60x objectives. Optimal microscope light settings were determined using positive and negative controls.

### Patient sera

In total, 103 sera from IBM patients were used. Normal healthy sera (NHS; n=34) were obtained from Sanquin blood bank. Informed consent was obtained from all patients from whom sera were used. The study protocol was in accordance with the Helsinki Declaration, and all procedures were approved by the local ethics committees.

### cN1A peptides

Four synthetic cN1A-derived 29-amino acid peptides (biotinylated at the C-terminus) were used to investigate the reactivity of the patient sera (219-DESE**R**IVKAHGLDRFFEHEKAHEN**K**PLAQ-247, synthesized by the LUMC peptide facility). The peptides differed in the monomethylation state. The control peptide did not have any monomethylated amino acids. Two peptides had one methylated amino acid, either at arginine-223 or lysine-243. The fourth peptide had monomethylated amino acids at both positions 223 and 243.

### ELISA

The ELISA was performed as described before, with some small adjustments.^31^ All incubation steps were performed for 1 hour at 37 °C. During all washing steps, plates were washed five times with PBS, 0.05% Tween-20 (PBST) using a plate washer (Nunc-Immuno Wash 8). Streptavidin-coated plates (Thermo Scientific™) were incubated with 30 ng biotinylated peptide per well in PBS containing 0.1% BSA. After peptide immobilization, wells were incubated with patient sera, 400-fold diluted in PBST, 1% BSA, and with rabbit-anti-human-Ig-HRP (1:2000, P0212, Dako), rabbit-anti-human-IgG-HRP (1:2000, P0214, Dako), or rabbit-anti-human-IgM-HRP (1:2000, P0215, Dako). TMB (3,3′,5,5′-tetramethylbenzidine) substrate in phosphate citrate buffer was added, and plates were incubated in the dark. After 10 min, H_2_SO_4_ was added to stop the reaction. The absorbance at 540 nm was subtracted from the absorbance at 450 nm (Varioskan LUX, Thermo Fisher). The average of the healthy subjects plus two times the standard deviation was used to determine the cut-off value. Graphs were made with GraphPad Prism 10.1.1 Data were analyzed using the Friedman test in GraphPad Prism.

## Results

### Identification of post-translational modifications in human skeletal muscle cN1A

**Figure 1.**
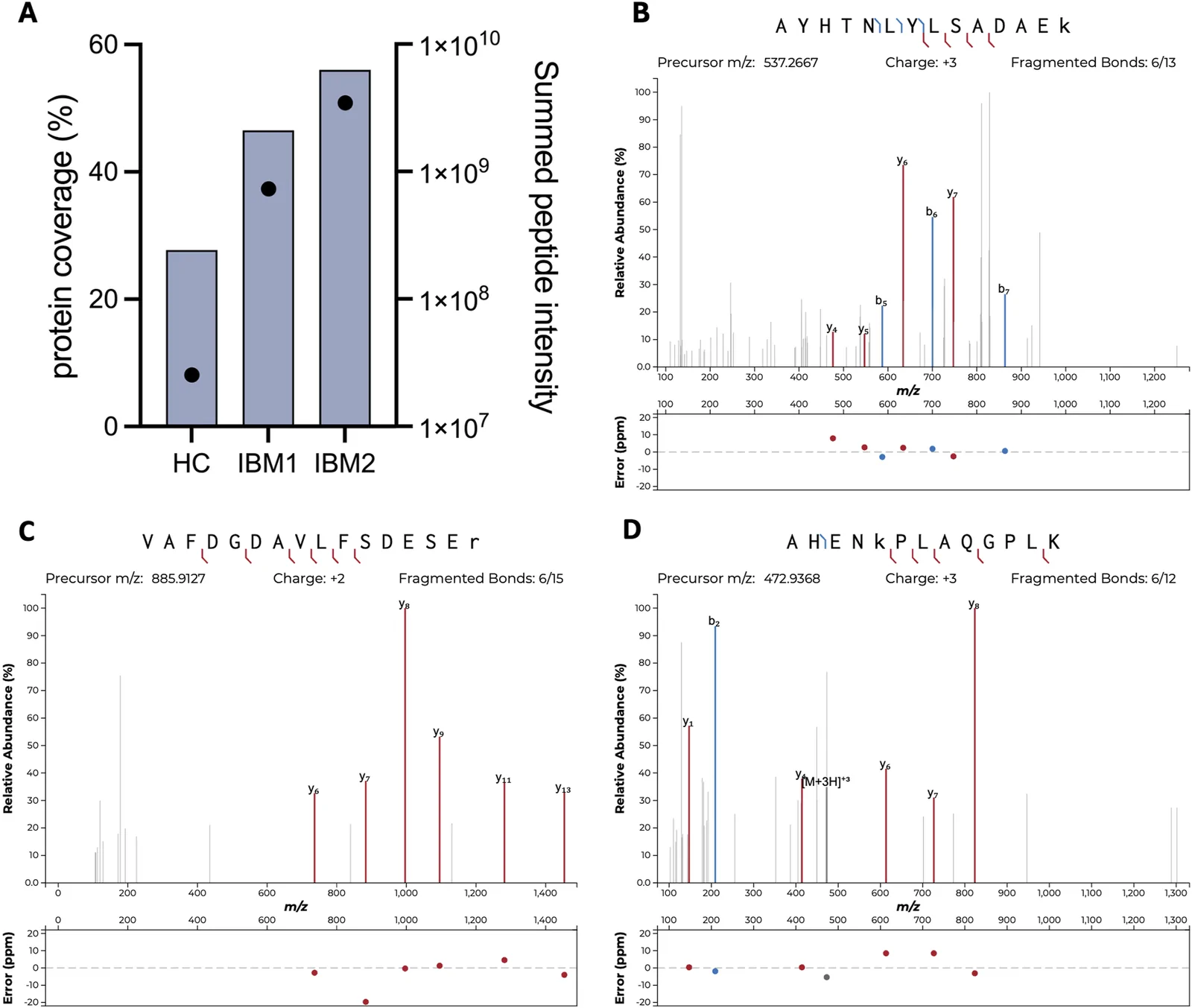
Identification of monomethylation sites of cN1A. cN1A isolated from human skeletal muscle tissue using antibodies from healthy control (HC) and IBM (IBM1 and IBM2) sera was digested with trypsin and subjected to mass spectrometry. (A) cN1A sequence coverage (bars) by peptides identified and summed peptide intensities (dots). (B–D) Annotated MS2 spectra of the peptides containing methylated residues; K178, R223 and K243, respectively (modified residues in sequences: lower case). b- and y-ions are marked in blue and red, respectively.

To get more insight into the post-translational modification of cN1A in skeletal muscle cells, cN1A was isolated from human skeletal muscle extracts and subjected to mass spectrometry. Polyclonal anti-cN1A autoantibodies from two IBM patient sera were used for immunoaffinity purification. As a control, antibodies from a healthy person’s serum devoid of anti-cN1A autoantibodies were used in parallel. Indeed, western blot analysis demonstrated that a polypeptide with an apparent molecular weight of 44,000 was isolated with the antibodies from the patient sera, but not with those of the healthy control (Supplementary Figure 1). The gel slices containing cN1A were excised from the gel, and after an in-gel digestion with trypsin, the polypeptides were analyzed by LC-MS/MS. The samples from the IBM patients resulted in the identification of 17 and 18 unique cN1A peptides, respectively. Although there was no visible cN1A band in the western blot, 14 unique cN1A peptides were identified in the healthy control sample. Nevertheless, Figure 1(a) shows that the peptide coverage of the healthy control (27.7%) is lower compared to antibodies from both IBM patients (46.5% and 56.0%). Moreover, the summed peptide intensity of cN1A in both IBM samples (7.3*10^8^ and 3.5*10^9^) was approximately 30- to 140-fold higher than that of the healthy control (2.5*10^7^). To screen for PTMs in an unbiased fashion, we initially conducted open database searches, which revealed a high frequency of peptide-spectrum matches (PSMs) with a mass shift (Δ mass) of 14.0156, indicative of monomethylation. Subsequent refined (closed) searches confirmed three specific monomethylation sites on cN1A: K178, R223, and K243 (Figure 1(b)–(d)). The monomethylated peptides occurred in 0.32% for K178, 0.62% for K243, and 0.29% for R223. The data did not indicate other common PTMs for cN1A (Supplementary Table 1).

### Effect of methylation site mutation on the subcellular accumulation of cN1A

**Figure 2.**
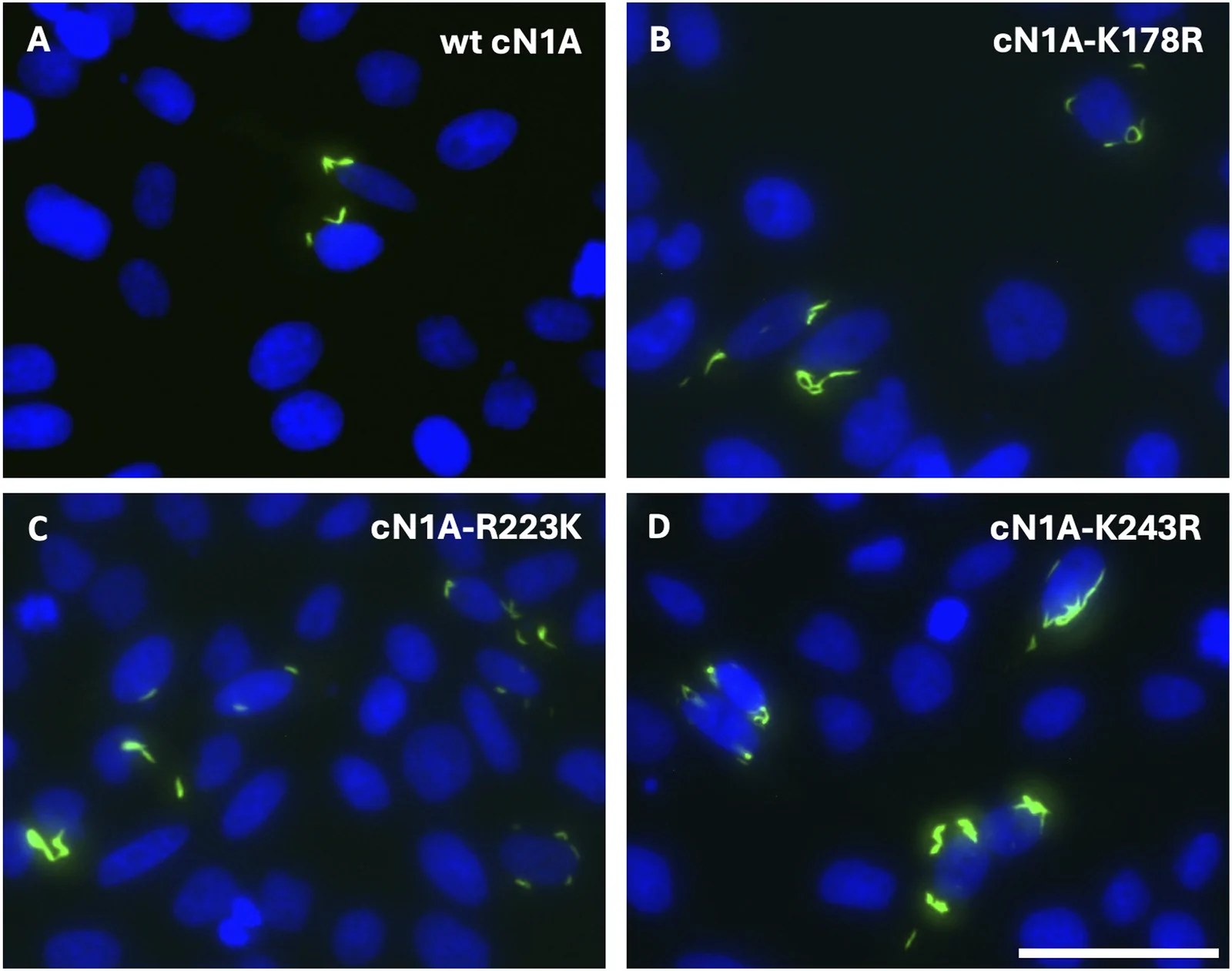
Subcellular localization of cN1A and cN1A methylation site substitution mutants in HEp-2 cells. (A) HEp-2 cells were transfected with a construct encoding the GFP-cN1A fusion protein. (B–D) HEp-2 cells were transfected with GFP-cN1A mutants, K178R, R223K, and K243R, respectively. Fluorescence microscopy was applied to visualize the localization of the (mutant) cN1A fusion protein (green). Nuclei were stained with DAPI (blue). The scale bar represents 100 μm.

Previously, we observed that ectopically expressed cN1A forms accumulations in the perinuclear region of cultured cells, as illustrated for HEp-2 cells in Figure 2(a). To investigate whether monomethylation influences the ability of cN1A to accumulate in perinuclear filaments, amino acid substitution mutants were generated. In these mutants, a lysine at the position of a monomethylated site is substituted by an arginine and vice versa. Since the physicochemical properties of lysine and arginine are similar, effects on cN1A accumulation are most likely due to the loss of monomethylation rather than to a structural change in cN1A resulting from the loss of a positive charge at these positions. Figure 2(b)–(d) shows the subcellular localization of mutant, N-terminally GFP-tagged cN1A in transfected HEp-2 cells. All mutants showed filamentous accumulations in the perinuclear region, which are comparable to the filaments observed for wild-type cN1A. These results suggest that cN1A monomethylation is not required for the accumulation of cN1A in perinuclear filaments in cultured cells.

### Effect of cN1A monomethylation on its recognition by autoantibodies

**Figure 3.**
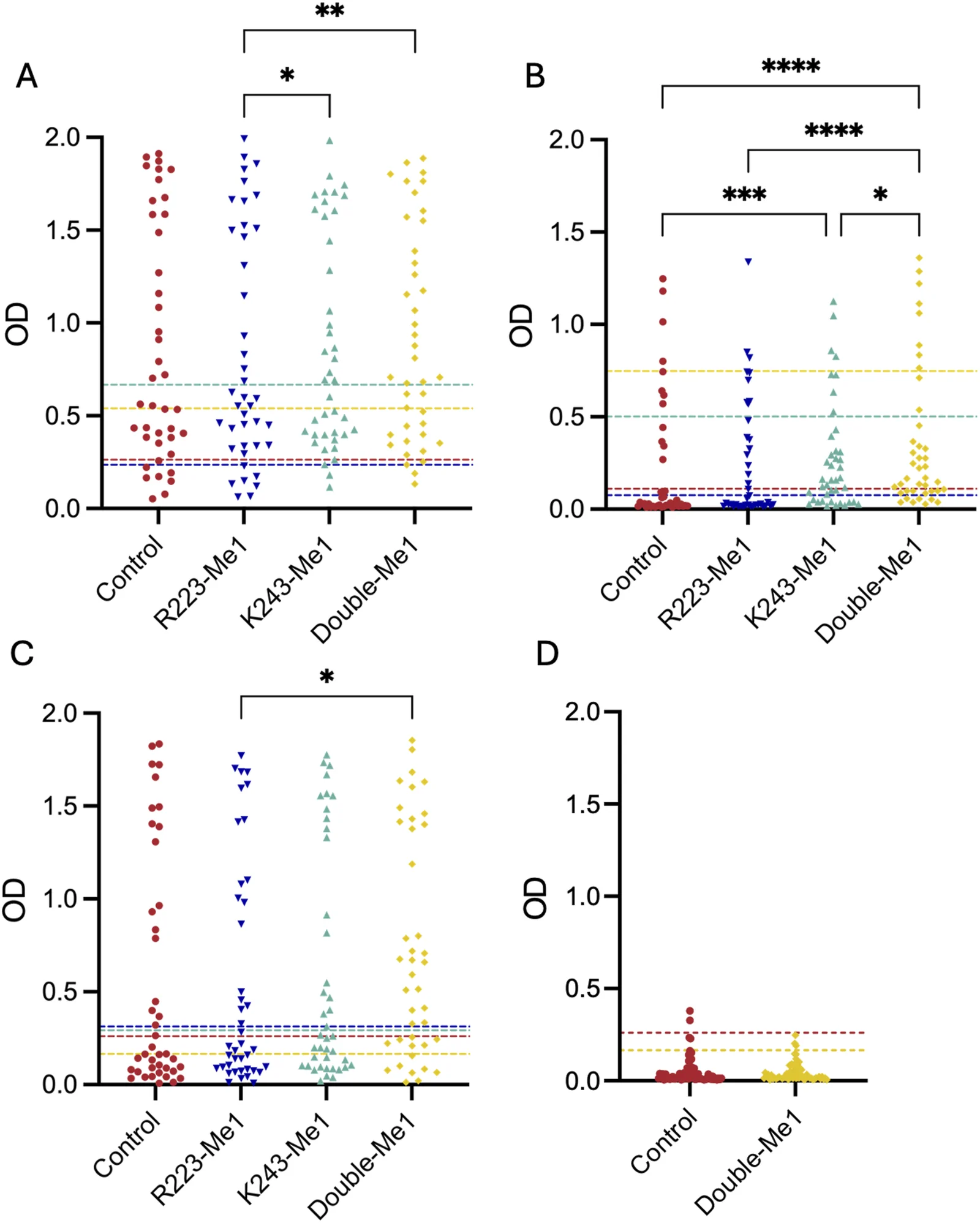
Recognition of (monomethylated) cN1A peptides by IBM patient sera. Streptawell plates were coated with biotinylated synthetic cN1A-derived peptides (aa219 – aa247) containing no (Control, red), one (R223-Me1, blue; K243-Me1, green), or two (R223 and K243, Double-Me1, yellow) monomethylated residues. The dashed lines represent the cut-off values for each peptide. Antibody-binding visualized by HRP-labeled anti-human IgG, IgA, and IgM (A), anti-human IgM (B), or anti-human IgG (C and D). (A–C) IBM patients (n=41), preselected based on reactivity with peptide 221-243. (D) IBM patients (n=62), previously shown not to be reactive with peptide 221-243.^34^ * p < 0.05, ** p< 0.01, *** p < 0.001, and **** p < 0.0001.

Two of the identified monomethylation sites, R223 and K243, lie within one of the three previously identified major linear epitopes of cN1A, aa 221-243.^7^ To investigate whether methylation influences the reactivity of anti-cN1A autoantibodies with this epitope, an ELISA was performed with synthetic peptides containing monomethylated residues at the respective positions. All peptides had the same amino acid sequence, corresponding to aa 219-247 of human cN1A, but they contained either no (control), one (R223-Me1 or K243-Me1), or two monomethylated residues (R223 and K243; double-Me1). Forty-one IBM patient sera were selected based on their reactivity with this epitope.^31^ Figure 3(a) shows the reactivity of anti-cN1A autoantibodies in these IBM patients with these four peptides. Although the sera were selected for their reactivity towards peptide 221-243, only 80% of these samples appeared to yield OD-values above the cut-off value with the 219-247 control peptide. While a similar fraction (83%) was reactive with peptide R223-Me1, peptides K243-Me1 and the double-Me1 were recognized by 54% and 66% of these sera, respectively (Table 1). These lower percentages seemed to be related to the higher cut-off values for these peptides (indicated by the dashed lines in Figure 3(a)). Previously, it has been reported that healthy subjects can have IgM autoantibodies reactive with monomethylated lysine residues.^32,33^ This raised the question of whether the presence of antibodies reactive with monomethylated lysine might influence the results obtained with the monomethylated lysine-containing peptides. These autoantibodies are also expected to be present in the samples from the healthy subjects, which were used to determine the cut-off values.

**Table 1.** Reactivity of preselected anti-cN1A-positive IBM patient sera with (monomethylated) cN1A 219-247 peptide.

%^a^	Control	R223-Me1	K243-Me1	Double-Me1
IgA, IgG & IgM	80	83	54	66
IgM	29	44	20	20
IgG	46	44	46	80

^a^Percentages of anti-cN1A autoantibody reactivity in IBM patient sera, detected with anti-IgA, IgG, IgM, anti-IgM, and anti-IgG secondary antibody-conjugates.

To investigate this possibility, a similar ELISA was performed but now using two distinct secondary antibodies: one targeting only human IgG antibodies (Figure 3(c)) and one specific for human IgM antibodies (Figure 3(b)). Table 1 shows the percentage of patients with reactivities above the cut-off value. Although the reactivity of the IgM autoantibodies reactive with the double methylated peptide is significantly higher compared to the other peptides, fewer patients are considered to have IgM autoantibodies reactive with the monomethylated lysine peptides (both 20%) compared to the other two peptides (29% and 44%, respectively). The lower percentages indeed seem to be due to the higher cut-off values resulting from the presence of IgM anti-monomethylated lysine antibodies, which may mask low-level anti-cN1A antibodies. As expected, with the IgG-specific secondary antibodies, the cut-off values for the peptides containing monomethylated lysine were substantially lower than with the anti-IgA, -IgG, -IgM as well as the anti-IgM secondary antibodies, substantiating the presence of IgM anti-monomethylated lysine antibodies. For IgG anti-cN1A autoantibodies, the percentage of patients reactive with the double-Me1 peptide (80%) was higher than for the other peptides (46%, 44%, and 46%, respectively), although this only reached statistical significance for the R223-Me1 peptide. This indicates that the use of the double monomethylated peptide might enhance the sensitivity of IgG anti-cN1A autoantibody detection.

To investigate whether screening for IgG autoantibodies with the double monomethylated peptide indeed increases the sensitivity, sera from 62 additional IBM patients were analyzed. In contrast to the 41 IBM patient samples preselected based on their reactivity with the non-methylated epitope, the additional sera previously did not show reactivity with peptide 221-243.^31^ We hypothesized that increased sensitivity as a result of the monomethylated residues might lead to a number of anti-cN1A-positive samples in this group. Figure 3(d) shows the reactivity of IgG autoantibodies of these 62 IBM patients with the control peptide and the double-Me1 peptide. Two of these sera showed weak reactivity with the non-methylated peptide 219-247, and 3 with the double-Me1 peptide. These results suggest that the use of the double methylated 219-247 peptide does not lead to a substantial increase in the frequency with which anti-cN1A is detected in IBM patient sera.

## Discussion

Previously, only a single type of PTM has been described for cN1A, phosphorylation in cancer cells.^27,28^ Here, we identified PTMs of cN1A in healthy skeletal muscle tissue, which is important for a better understanding of the role of cN1A in normal muscle fibers and in the pathogenesis of IBM. In addition, we investigated whether the identified monomethylation of cN1A affects its subcellular distribution and its recognition by autoantibodies.

The characterization of human muscle cN1A by mass spectrometry resulted in the identification of three monomethylation sites, located at K178, R223, and K243. The identified sites exhibited strong localization confidence (>95% probability via PTMProphet), further supported by manual diagnostic fragment ion verification. Low levels of the peptides were monomethylated, which suggests that only a minor fraction of cN1A is methylated in healthy human muscle tissue. Targeted isolation of the 44 kDa cN1A band provided the analytical sensitivity required to capture these low-abundance proteoforms, while unconstrained open database searching cleanly isolated the +14.0156 Da monomethylation peak, ruling out non-specific chemical misassignments. Since side-chain monomethylation can theoretically introduce steric hindrance that limits tryptic digestion efficiency, the calculation of occupancy and quantification of modification sites can be inherently limited if the corresponding unmodified peptides are not consistently detected in the exact same cleavage state. Interestingly, our mass spectrometric data revealed that two of the identified monomethylated peptides (K178 and R223) were predominantly detected as fully cleaved, tryptic forms, making the quantification and occupancy calculation of these respective sites less problematic. In contrast, for the K243 site, only the missed-cleavage variants of the unmodified peptides were detected, which may inherently limit the absolute stoichiometric precision. These values should therefore be interpreted as a conservative baseline representation of cN1A methylation frequency.

Moreover, low fractional stoichiometry is known for PTMs, e.g. acetylation in non-histone cytosolic signaling and regulatory modifications in steady-state tissue.^35^ Even at low levels, acetylation has been demonstrated to be functionally relevant. Two lysine residues of DNA methyltransferase 1 (DNMT1) have acetylation levels of 0.57% and 0.1% in HeLa cells, respectively.^35^ Acetylation of DNTM1 is known to trigger proteasomal degradation.^36^ The activity of end-binding protein 1 (EB1) during mitosis is also regulated by acetylation.^37^ Only 0.02% of EB1 was found to be acetylated in HeLa cells,^35^ but during mitosis, EB1 acetylation levels were found to be 4 times higher.^37^ Phosphoglycerate kinase 1 (PGK1) acetylation inhibits the binding of ADP, thereby disrupting its enzymatic activity, which can be reversed by insulin signaling.^38^ The acetylation level of PGK1 in HeLa cells is only 0.03%.^35^ Therefore, even though the methylation levels of cN1A are low, it might still be biologically relevant.

The amino acid sequence elements flanking the identified methylation sites in cN1A were compared with the substrate recognition motifs of a number of lysine and arginine methyltransferases. The available information on methylation sites for the lysine methyltransferases ATXR5, Cl4, Dim-5, Efm1, G9a, NSD1, SET7/9, SET8, SUV39H1, SUV39H2, SUV4−20H1, and SUV4−20H2^39^ and for the arginine methyltransferases Hmt1, PRMT1,^39^ PRMT7,^40^ did not reveal high levels of similarity, suggesting that these enzymes are not involved in cN1A methylation. However, it should be noted that alternative recognition sequences, compared to consensus motifs, have been reported for several protein methyltransferases and, therefore, we cannot completely rule out the involvement of these methyltransferases.

Here, we have identified the methylation levels of cN1A in healthy muscle tissue. It remains to be elucidated whether (patho)physiological changes, e.g., inflammation in the affected muscles in IBM patients, affect cN1A’s methylation levels. Based on the present data, we can also not conclude the co-occurrence of these methylations on a single molecule of cN1A. In addition, the potential relationship with phosphorylation remains to be investigated.

Substitution of the monomethylated amino acids did not detectably affect the accumulation of cN1A in perinuclear filamentous structures in cultured HEp-2 cells. This indicates that monomethylation of these residues is not required for the accumulation behavior of cN1A. Because the monomethylation levels of the wild-type cN1A in transfected cultured cells are unknown, the possibility exists that methylation at one or more of these sites interferes with filament formation. If these levels are low, like in muscle fibers, the effects of methylation on cN1A’s subcellular accumulation, as assessed by analysis of substitution mutants, might have escaped our attention. Another limitation of the lysine to arginine and arginine to lysine substitutions is that differences in the physicochemical and biochemical properties of lysine and arginine imply that any observed phenotype (or lack thereof) may be due to other alterations than (solely) the loss of methylation. It is also important to recognize that the epithelial HEp-2 cells do not represent the most optimal cell line to study cell biological aspects of cN1A. Although technically more challenging, using cultured human myotubes should be considered to substantiate these data.

Methylation^20^ and ubiquitination^21^ of CTPS have been shown to affect its filament formation and to influence its enzymatic activity. Although CTPS remains active when it assembles into tetramers, this activity is reduced when these tetramers form filaments. Since methylation can regulate filament formation,^20^ PTMs influence the activity of the enzyme. It remains to be investigated whether cN1A is enzymatically active in filamentous structures and whether monomethylation influences its activity.

The analysis of (monomethylated) synthetic peptides corresponding to one of the major previously identified linear epitope regions of cN1A demonstrated increased IgG autoantibody reactivity with the peptide containing monomethylated residues at both aa 223 and aa 243 (80%) compared to the non-methylated and single monomethylated counterparts (∼45%) for preselected IBM sera reactive with this epitope. Additional analyses with sera that previously did not show reactivity with this epitope^31^ did not lead to additional support for the increased antigenicity by methylation of both residues, although it should be noted that the number of reactive sera in this group was low. The tested serum samples were selected based on their (lack of) reactivity in a similar peptide ELISA.^31^ In our experiments, the non-methylated peptide was similar, though not completely identical to the peptide used by Herbert and coworkers, because it was extended by 2 and 4 amino acids at the N-terminus and C-terminus, respectively. This difference may, at least in part, explain why only 80% of the preselected positive sera appeared to be reactive with the non-methylated peptide.

During the experiments, we noticed that the cut-off values were higher for monomethylated lysine (K243)-containing peptides than for the non-methylated peptide and for the monomethylated arginine-containing peptide. The cut-off values are based on the results obtained with samples from healthy individuals. The higher cut-off values are most likely explained by IgM autoantibodies targeting methylated lysine residues, which also occur in healthy subjects, as reported previously.^32,33^ These antibodies specifically react with monomethylated lysine residues and not with non-modified or di- or trimethylated lysine residues. Experiments with immunoglobulin isotype-specific secondary antibodies indeed confirmed that the relatively high cut-off values were consistent with the frequent presence of IgM anti-monomethylated lysine antibodies in human sera. The potential presence of IgM anti-monomethylated lysine antibodies should be considered during the interpretation of the data when IgM-reactive secondary antibodies are used.

To conclude, we identified three monomethylation sites in cN1A from healthy human skeletal muscle. These methylation sites do not seem to influence the capability of cN1A to form filaments in cultured cells. However, the simultaneous monomethylation of R223 and K243 may lead to a more frequent recognition of the most central linear autoepitope (221-243) of cN1A by IBM patient sera.

## Supplemental material

Supplemental material - Monomethylation of cytosolic 5′-nucleotidase 1A affects its recognition by inclusion body myositis autoantibodies Supplemental material for Monomethylation of cytosolic 5′-nucleotidase 1A affects its recognition by inclusion body myositis autoantibodies by Fleur Brinkman, Marian Barandica Graciani, Amrah Weijn, Filine Swets, Seher Al Bana, Chien-Yun Lee, Ger J. M. Pruijn in Journal of Neuromuscular Diseases

Supplemental material - Monomethylation of cytosolic 5′-nucleotidase 1A affects its recognition by inclusion body myositis autoantibodies Supplemental material for Monomethylation of cytosolic 5′-nucleotidase 1A affects its recognition by inclusion body myositis autoantibodies by Fleur Brinkman, Marian Barandica Graciani, Amrah Weijn, Filine Swets, Seher Al Bana, Chien-Yun Lee, Ger J. M. Pruijn in Journal of Neuromuscular Diseases

## Data Availability

The mass spectrometry proteomics data have been deposited to the ProteomeXchange Consortium via the PRIDE partner repository^41^ with the dataset identifier PXD075326.
