# Supplementary material for: Monomethylation of cytosolic 5′-nucleotidase 1A affects its recognition by inclusion body myositis autoantibodies
Source: J Neuromuscul Dis. 2026 Sep 2:22143602261487219. Online ahead of print. doi: 10.1177/22143602261487219 (PMC13538286; doi:10.1177/22143602261487219)
Supplement: Supplementary file 1 [file sj-pdf-1-jnd-10.1177_22143602261487219.pdf]

Supplementary material

Monomethylation of cytosolic 5'-nucleotidase 1A affects its recognition by inclusion body myositis autoantibodies

Fleur Brinkman, Marian Barandica Graciani, Amrah Weijn, Filine Swets, Seher Al Bana, Chien-Yun Lee, Ger J.M. Pruijn

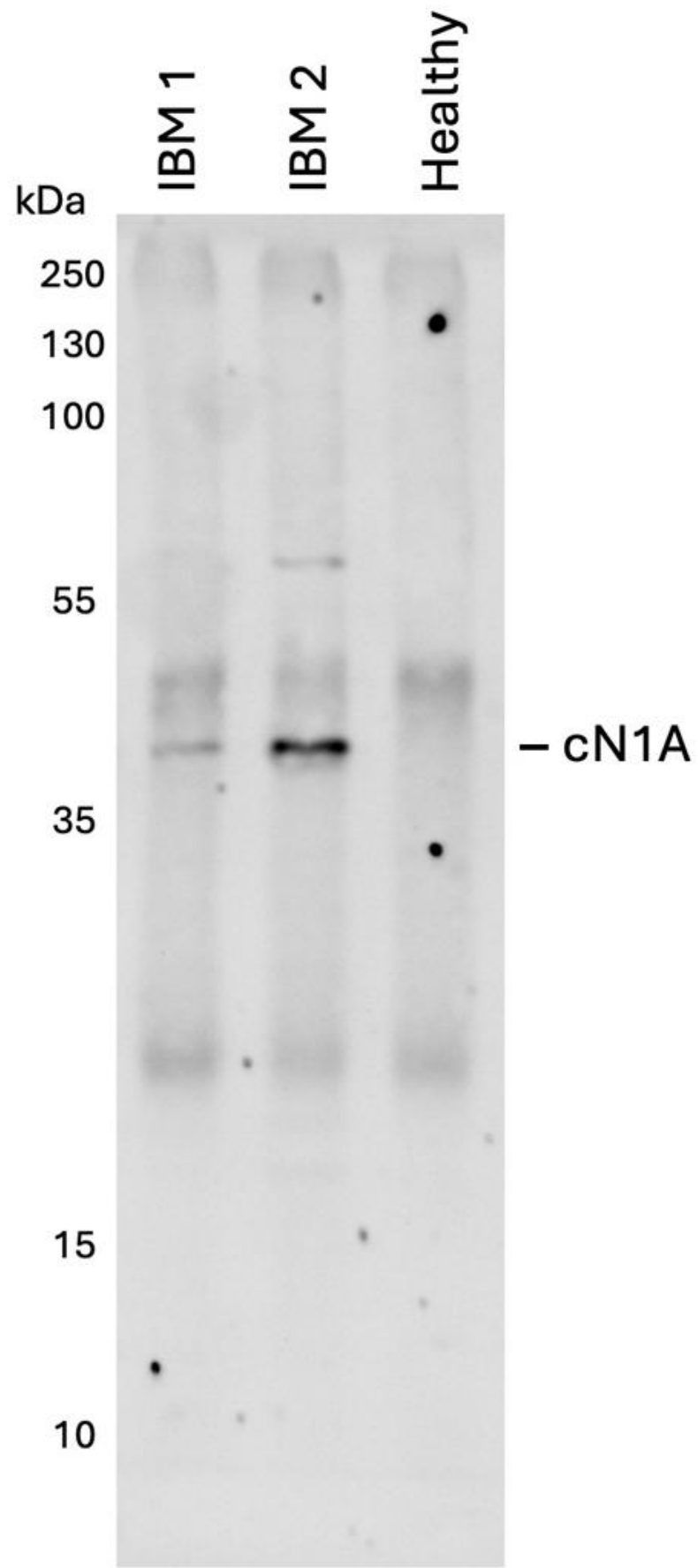

Supplementary Figure 1: Isolation of cN1A from skeletal muscle tissue. Antibodies from three sera, two from IBM patients (IBM 1 and IBM 2) and one from a healthy person, were used to isolate cN1A from tissue lysates by immunoaffinity chromatography. The isolated proteins were analyzed by western blotting and staining with the serum of IBM patient 2. The position of molecular weight markers is indicated on the left.

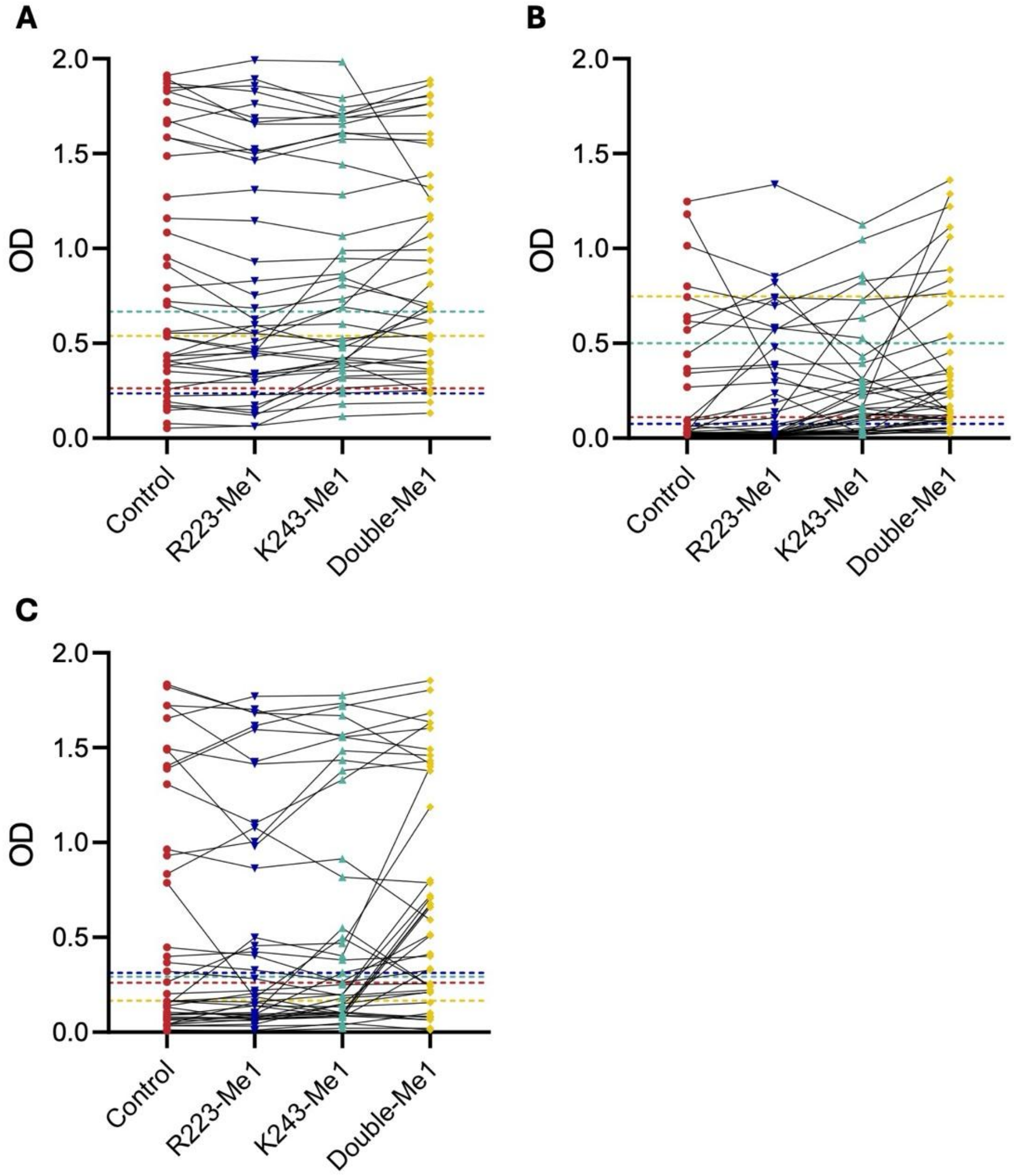

<u>Supplementary Figure 2:</u> Recognition of (monomethylated) cN1A peptides by individual IBM patient sera. The reactivity of preselected IBM patients' sera (n=41; preselected based on reactivity with peptide aa 221–243) with cN1A-derived synthetic peptides (aa 219–247), containing no (Control, red), one (R223-Me1, blue; K243-Me1, green), or two (R223 and K243, Double-Me1, yellow) monomethylated residues was analyzed by ELISA. The lines mark the results obtained with individual patient sera. The colored dashed lines represent the cut-off values for the corresponding peptides. Antibody-binding was visualized by HRP-labeled anti-human IgG, IgA, IgM (A), anti-human IgM (B), or anti-human IgG (C).
